# Heterogeneous zonal impacts of climate change on a wide hyperendemic area of human and animal fascioliasis assessed within a One Health action for prevention and control

**DOI:** 10.1371/journal.pntd.0012820

**Published:** 2025-01-21

**Authors:** Pablo Fernando Cuervo, María Dolores Bargues, Patricio Artigas, Paola Buchon, Rene Angles, Santiago Mas-Coma

**Affiliations:** 1 Departamento de Parasitología, Facultad de Farmacia, Universidad de Valencia, Burjassot, Valencia, Spain; 2 CIBER de Enfermedades Infecciosas, Instituto de Salud Carlos IIII, Madrid, Spain; 3 Unidad de Limnología, Instituto de Ecología, Universidad Mayor de San Andrés (UMSA), La Paz, Bolivia; 4 Cátedra de Parasitología, Facultad de Medicina, Universidad Mayor de San Andrés (UMSA), La Paz, Bolivia; IRNASA, CSIC, SPAIN

## Abstract

The Northern Bolivian Altiplano is the fascioliasis endemic area where the highest prevalences and intensities in humans have been recorded. In this hyperendemic area of human fascioliasis, the disease is caused only by *Fasciola hepatica* and transmitted by *Galba truncatula*, the sole lymnaeid species present in the area. When analysing the link between global warning and the recently reported geographical spread of lymnaeid populations to out-border localities, a marked heterogeneous climatic change was found throughout the endemic area. The aim of the present study was to analyse the physiographical heterogeneity of the fascioliasis hyperendemic area in the Northern Bolivian Altiplano, in order to assess its repercussions in the implementation of a One Health action. We applied multivariate linear mixed models to analyse the influence of a number of physiographical features on the long-term variation of climate and of the risk of transmission. Despite its apparent physiographic homogeneity, the findings of this study revealed markedly heterogeneous climate characteristics throughout the endemic area. This irregular pattern is influenced by physiographical features such as altitude, inner hills, closeness to Lake Titicaca, and El Niño–Southern Oscillation. This is the broadest study ever performed in a human fascioliasis endemic area about the influence of physiography on climate. It highlights the importance of considering physiographical features, an aspect usually not considered in studies dealing with the influences of climate and climate change on human and animal fascioliasis. Moreover, it shows that an endemic area may climatically evolve differently in its various inner zones and emphasizes the need for continuous monitoring to assess whether control measures should be modified accordingly.

## Author summary

Fascioliasis is a snail-borne zoonotic disease caused by the trematodes *Fasciola hepatica* and *Fasciola gigantica*. The highest prevalences and intensities ever found in humans have been reported in the Northern Altiplano of Bolivia, in South America. In this endemic area, the disease is caused only by *Fasciola hepatica* and transmitted by *Galba truncatula*, the sole lymnaeid species present in the area. Our aim was to analyse the physiographical heterogeneity of this fascioliasis hyperendemic area, and assess its repercussions in the implementation of a One Health action. We applied multivariate linear regressions to analyse the influence of physiographical features on the long-term variation of climate and of the transmission risk. Our results revealed markedly heterogeneous climate characteristics throughout the endemic area. This irregular pattern is influenced by altitude, closeness to inner hills and Lake Titicaca, and El Niño–Southern Oscillation. These findings highlight the importance of considering physiographical features, an aspect usually not considered in studies dealing with influences of climate and climate change on human and animal fascioliasis. Moreover, it shows that an endemic area may climatically evolve differently in its various inner zones and emphasizes the need for continuous monitoring to assess whether control measures should be modified accordingly.

## 1. Introduction

Fascioliasis is a freshwater snail-borne zoonotic disease caused by two trematode species, *Fasciola hepatica* and *F. gigantica*, which affects humans and herbivorous mammals, mainly livestock. This disease is well-known in the veterinary field, as it causes high losses in husbandry worldwide, above all of cattle and sheep [1]. From the public health point of view, human fascioliasis has become an emerging public health problem (e.g., [2]). Endemic areas have been reported in many countries and the number of human case reports is progressively increasing [3]. This worrying scenario adds to its high pathogenicity [4,5], potential long-term post-treatment sequelae [6], and the immunosuppression in both the acute and chronic phases of the disease [7–9]. The latter underlies usual coinfections with other pathogenic protozoans and helminths leading to high morbidity [10,11], and even mortality, in hyperendemic areas of mainly low-income but also developed countries [12]. According to all this, the World Health Organization (WHO) categorized fascioliasis within the Foodborne Trematodiases listed as priorities among the Neglected Tropical Diseases (NTDs) in its WHO NTD Roadmaps for 2020 and 2030 [13,14]. Moreover, WHO has very recently underscored the convenience of applying a comprehensive One Health approach to attain the targets outlined in the Roadmaps [15].

The Northern Bolivian Altiplano is the fascioliasis endemic area where the highest prevalences and intensities in humans have been recorded [16–18], rising to 72% and 100% prevalence by coprology and serology according to localities, respectively [19]. Children are the most affected, becoming infected very early in their lives, with more than 3000 eggs per gram of feces (epg) [10], even reaching up to 8000 epg [20]. In this hyperendemic area of human fascioliasis, the disease is caused only by *F. hepatica* and transmitted by *Galba truncatula*, the sole lymnaeid species present in the area [21,22]. Both parasite and snail intermediate host have been introduced from Europe by the Spanish “conquistadores” [23]. Given the very high infection risk in this hyperendemic area and with the purpose of alleviating the situation, the WHO launched a preventive chemotherapy strategy by means of yearly mass treatment campaigns [20,24] implemented through a multidisciplinary One Health action [19].

The aim of the present study is to describe the marked heterogeneous climatic change found throughout the endemic area when analysing the link between global warning and the recently reported geographical spread of lymnaeid populations in the Bolivian Altiplano [22,25]. This climatic heterogeneity is worth mentioning because of (i) its influential impact on fascioliasis in this hyperendemic area and (ii) despite the a priori counteracting homogenous physiography of the flatland corridors throughout which the disease endemic is transmitted and consequently distributed. Although previous studies emphasize the closeness to Lake Titicaca or the Oriental Andean Chain as influential factors on the area in question [26], and thus on the local fascioliasis transmission [17], these factors alone do not seem to account for the high variability we have verified at the local scale.

Our study is a deep analysis which allows to highlight a real physiographical heterogeneity of the Northern Bolivian Altiplano hyperendemic area and the influences it underlies on the long-term evolution of climate and disease transmission risk. The focus is arranged in the way to assess the repercussions of the physiographical features of both inside the endemic area and its circum-surrounding zones in the implementation of a One Health action and has therefore a great interest for potential extrapolation to and hence usefulness for other endemic areas.

## 2. Methods

### 2.1. Study area

**Fig 1:**
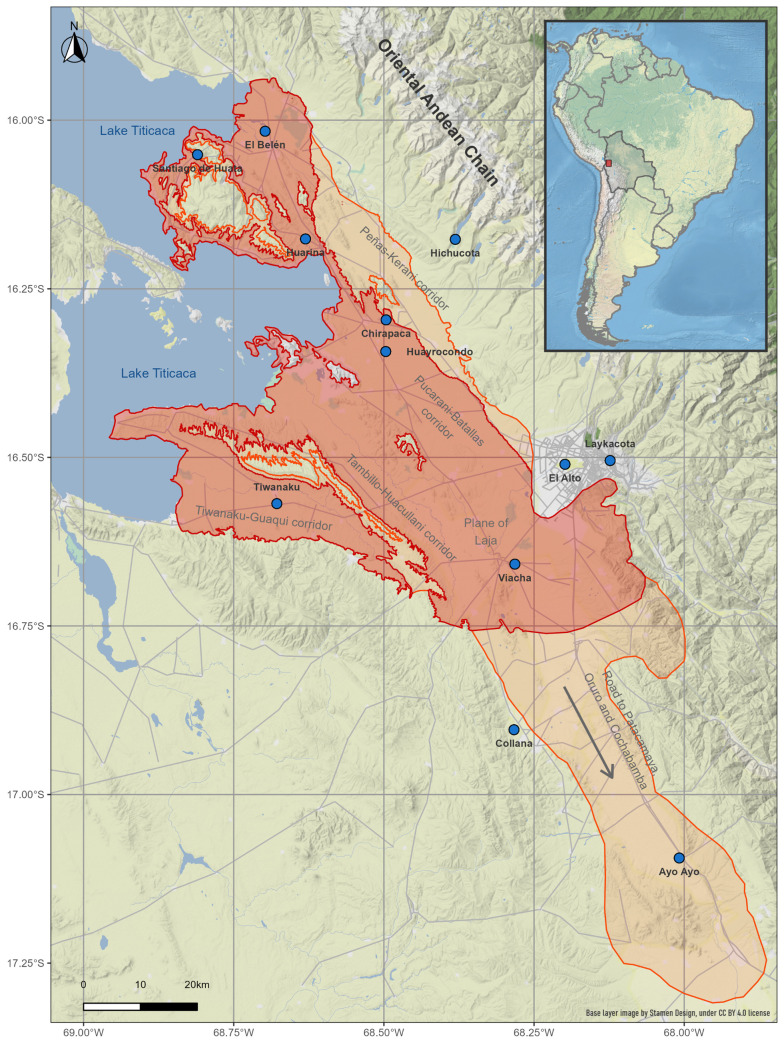
Study area in the Northern Bolivian Altiplano human fascioliasis hyperendemic area. The map shows the meteorological stations included in the study (blue circles, detailed in Table 1). Former endemic area defined throughout the 1990’s [17], in red; and current endemic area, in orange (for further details see [22,27]). Base layer image by Stamen Design, under CC BY 4.0 license (https://maps.stamen.com/), roads shapefile from Natural Earth are in the public domain (https://www.naturalearthdata.com/about/terms-of-use/), and countries border shapes from GADM are freely available for academic use and other non-commercial use (https://gadm.org/license.html).

The study was focused on the Northern Bolivian Altiplano human fascioliasis hyperendemic area (Fig 1). This area is located between Lake Titicaca and the valley holding the city of La Paz (14–17.5° S, 67.5–71° W), at an altitude ranging between 3800 and 4100 m above sea level [17]. The endemic area covers the Northern Altiplano, also known as humid Altiplano, including part of Los Andes, Ingavi, Omasuyos and Murillo provinces of the Department of La Paz [27]. Most of it concerns the two large corridors (planes separated by small hill chains) El Alto-Pucarani-Batallas and Tambillo-Aygachi-Huacullani, the plane of Laja in which both corridors reunite, and up to the route from El Alto to Oruro [17].

**Table 1:** Meteorological stations and respective time periods analysed in the region of the Northern Bolivian Altiplano where human fascioliasis is hyperendemic.

Station	Department	Province	Geographical coordinates	Altitude	Time period
a. Ayo Ayo	La Paz	Aroma	17° 05′ 39″ S–68° 00′ 30″ W	3888	1958–2020
b. Chirapaca	La Paz	Los Andes	16° 17′ 46″ S–68° 29′ 47″ W	3870	1991–2020
c. Collana	La Paz	Aroma	16° 54′ 01″ S–68° 16′ 54″ W	4500	1973–2020
d. El Alto	La Paz	Murillo	16° 30′ 37″ S–68° 11′ 55″ W	4071	1962–2020
e. El Belén	La Paz	Omasuyos	16° 00′ 59″ S–68° 41′ 52″ W	3833	1949–2017
f. Hichucota	La Paz	Los Andes	16° 10′ 36″ S–68° 22′ 52″ W	4460	1979–2020
g. Huarina	La Paz	Omasuyos	16° 10′ 34″ S–68° 37′ 50″ W	3838	1973–2011
h. Huayrocondo	La Paz	Los Andes	16° 20′ 35″ S–68° 29′ 49″ W	3875	1991–2020
i. Laykacota	La Paz	Murillo	16° 30′ 17″ S–68° 07′ 24″ W	3632	1945–2020
j. Santiago de Huata	La Paz	Omasuyos	16° 03′ 04″ S–68° 48′ 37″ W	3845	1985–2020
k. Tiwanaku	La Paz	Ingavi	16° 34′ 07″ S–68° 40′ 42″ W	3863	1973–2016
l. Viacha	La Paz	Ingavi	16° 39′ 30″ S–68° 16′ 55″ W	3850	1965–2015

### 2.2. Climatic data

Monthly climatic data from 12 meteorological stations located in the Northern Bolivian Altiplano were retrieved from the “*Servicio Nacional de Meteorología e Hidrología*” (http://senamhi.gob.bo/index.php/sismet, accessed September 2021). The climatic data provided covered a standard 30-year climatological reference period in every case, but covered longer periods in most cases. The meteorological stations included in this study are detailed in Table 1, and its geographical location in the human fascioliasis hyperendemic area of the Northern Bolivian Altiplano is depicted in Fig 1.

The monthly data analysed were mean environmental temperature (MET), mean maximum temperature (MMT), mean minimum temperature (MmT), extreme maximum temperature (EMT), and extreme minimum temperature (EmT), all in °C, precipitation (Pt), maximum precipitation (MP), total potential evapotranspiration (PET), all in mm, number of days with precipitation (DP) and number of days with frost (DF) [28]. In addition, yearly precipitation (YP) considered the mean precipitation cumulated throughout an entire year.

Similarities between meteorological stations were assessed visually with violin plots and statistically with analysis of variance followed by post-hoc Tukey’s test. Violin plots are somehow similar, but more informative than box plots, as they also show the full distribution of the data in the form of probability density [29].

### 2.3. Climatic forecast indices

The incidence of fascioliasis infection in the definitive host has been related to air temperature, rainfall and/or potential evapotranspiration [30,31]. These factors affect the intermediate snail host population dynamics and the parasite population at the level of both the free-living larval stages of egg and metacercaria and the intramolluscan parasitic larval stages of sporocyst, rediae and cercariae. Climatic fascioliasis forecast indices are calculated with different equations which consider variations in these climatic factors (i.e., [32–36]).

After introducing modifications for high altitude and low latitude, the two most useful indices have been previously applied in the Bolivian Altiplano [25,28]: the Wet Day index (Mt index) (proposed by Ollerenshaw and Rowlands [32], and modified by Ollerenshaw [33,34]) and the Water budget-based system index (Wb-bs index) (proposed by Malone et al. [35] and modified for large scale regional use [36]).

The Wet Day index (Mt) [32] is expressed by the equation:


$$Mt=\frac{n\left(R-PET+125\right)}{25}$$

where *n* is the number of rain days, *R* is the rainfall in mm, and *PET* is the potential evapotranspiration in mm [33,34]. For the calculation of this index, the only months considered are those in which the MET is ≥10°C, since this temperature is considered the lower threshold temperature for the development of fascioliasis by *F. hepatica* [32,37].

The Water-budget-based system (Wb-bs) [35], adapted for large scale regional application using monthly climatic data [36], is expressed as:


$$Wb-bs\;=\;\left(GDD\;*\;days\;\;in\;\;month\right),\;\;if\;\;\left[R-\left(PET\;*\;0.8\right)\right]>0,$$

$$+\;\left(GDD\;*\;n\right)\;*\;\frac{R-PET}{25},\;\;if\;\left(R-PET\right)>0$$

where *R* is the rainfall, *PET* the potential evapotranspiration, *n* the monthly number of days with surplus rainfall (>1 mm), and *GDD* the growing degree-days calculated as the monthly MET-10 °C [38], which is the minimum development temperature for *F. hepatica* [32,37]. In the first part of the formula, subtracting the factor (*PET* x 0.8) from rainfall (*R*) is assumed to be equivalent to counting monthly GDD if moisture storage is present in the top 2.5 cm layer of a soil water budget model. The second part counts GDD if monthly surplus water is present due to rainfall events [35,36].

Since climate diagrams furnished appropriate results on the duration of the wet and dry seasons only after introducing the modification of Schreiber (1981) [39] in the aridity calculation [31], the Mt and Wb-bs forecast indices were accordingly modified to account for high altitude and low latitude, as previously applied in the human fascioliasis hyperendemic area [25,28]. Potential evapotranspiration (PET) is replaced by Schreiber’s aridity index *r* [39] (named from now on as *AI*), calculated as follows:


AI=2tk+  0.03tk2  *  S12

where *t*_*k*_ is the corrected mean monthly temperature (which is increased by an altitude factor), and *S* is the mean monthly daylight in hours (which becomes increasingly noticeable at higher latitudes). Additionally, as the MmT (often corresponding to night-time temperatures) reached in a large part of the study area causes the MET to fall below of 10 °C for much of the year, the calculations were modified to give relevance to the MMT [40], which exceeds the minimum temperature required for the start of activity of the intermediate lymnaeid host and free-living stages of *F. hepatica* during long periods of the year.

Summarizing, the two indices were calculated according to the formulae proposed for high altitudes in tropical or subtropical areas [28]:


$$Mt=\frac{n\left(R-AI+125\right)}{25}$$

$$Wb-bs=\left(GDD\;*\;days\;\;in\;\;month\right),\;\;if\;\;\left[R-\left(AI\;*\;0.8\right)\right]\;>0,$$

$$+\;\left(GDD\;*\;n\right)\;*\;\frac{R-AI}{25},\;\;if\;\;\left(R-AI\right)>0$$

where *AI* is the aridity index, and *GDD* = [(MET + MMT)/2] – 10, considering only those months in which [(MET + MMT)/2] is ≥10°C.

Months giving a value for Mt equal to or higher than a critical value are considered potential high-risk periods for the incidence of the disease. Mt values sufficient to support transmission have been considered as ≥100 in UK, 80 in France [33,34], and as low as 55–60 in Pakistan [41].

The Wb-bs index was analysed on the basis of accumulative values in a continuous way when different from 0. Risk values conventionally established and used by several authors, are: 600 = no risk; 601–1500 = low risk; 1500–3000 = moderate risk; and 3000 = high risk [28,36,38,40–42].

### 2.4. Analysis of the influence of physiographical features and El Niño—Southern Oscillation (ENSO) on climatic factors and climatic forecast indices

**Fig 2:**
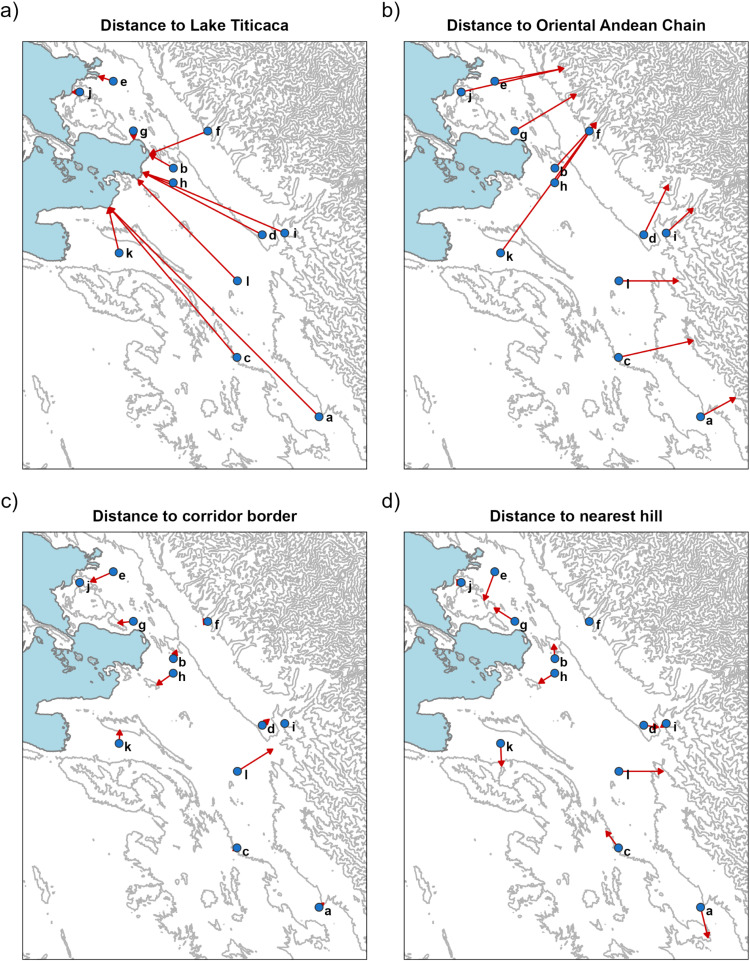
Shortest distances from the meteorological stations included in the study to physiographical features of interest: A) distance to Lake Titicaca; B) distance to the Oriental Andean Chain; C) distance to the nearer border of inter-hill corridors; D) distance to nearest hill. References of meteorological stations (see details in Table 1): a) Ayo Ayo; b) Chirapaca, c) Collana; d) El Alto; e) El Belén; f) Hichucota; g) Huarina; h) Huaycorondo; i) Laykacota; j) Santiago de Huata; k) Tiwanaku; l) Viacha. Polygon of the Lake Titicaca extracted from HydroLAKES under CC BY 4.0 license (https://www.hydrosheds.org/products/hydrolakes), and contour lines derived from a georeferenced 3 arc second (~90 m resolution) SRTM DEM from CGIAR-CSI under CC BY 4.0 license (https://csidotinfo.wordpress.com/data/srtm-90m-digital-elevation-database-v4-1/).

We analysed the influence on climatic factors and climatic forecast indices of a number of physiographical features and of El Niño–Southern Oscillation (ENSO). The physiographical features included in the analyses are detailed in Table 2 and distances are depicted in Fig 2. As a representation of the ENSO, we used the Multivariate ENSO Index (MEI), which depicts a more holistic surrogate of the atmospheric and oceanic anomalies that occur during ENSO events [43]. Monthly time-series of MEI (version 1), covering from 1950 to 2018, were retrieved from the NOAA Physical Sciences Laboratory (https://psl.noaa.gov/enso/mei.old/).

**Table 2:** Detail of the physiographical variables included in the models analysing the influence of geographical features and El Niño–Southern Oscillation (ENSO) on climatic factors and climatic forecast indices.

Acronym	Variable description	Processing procedure
dist2lake	Distance to Lake Titicaca	Shortest distance from meteorological station to polygon of the Lake Titicaca^1^
dist2contour	Distance to inter-hill corridor borders	Shortest distance from meteorological station to corridor borders, defined by contour lines derived from a Digital Elevation model (DEM)^2^
dist2slope	Distance to nearby hills	Shortest distance from meteorological station to areas with slope greater than 15°, defined from a ‘slope’ raster^3^
dist2Andes	Distance to the steepest hillsides of the Oriental Andean Chain	Shortest distance from meteorological station to areas with slope greater than 25°, defined from a slope raster^3^
altitude	Elevation derived from different high resolution DEMs	Values extracted for each meteorological station from ‘elevation’ raster by bilinear sampling^3^
northness	Sine of the slope, multiplied by the cosine of the aspect, describing the orientation in combination with the slope	Values extracted for each meteorological station from ‘northness’ raster by bilinear sampling^3^
eastness	Sine of the slope, multiplied by the sine of the aspect, describing the orientation in combination with the slope	Values extracted for each meteorological station from ‘eastness’ raster by bilinear sampling^3^
VRM	Vector Ruggedness Measure (VRM) quantifies terrain ruggedness by measuring the dispersion of vectors orthogonal to the terrain surface	Values extracted for each meteorological station from ‘VRM’ raster by bilinear sampling^3^

**Sources**

^1^Georeferenced polygon of the Lake Titicaca extracted from HydroLAKES under CC BY 4.0 license (https://www.hydrosheds.org/products/hydrolakes) [44].

^2^Georeferenced 3 arc second (~90 m resolution) SRTM DEM from CGIAR-CSI under CC BY 4.0 license (https://csidotinfo.wordpress.com/data/srtm-90m-digital-elevation-database-v4-1/).

^3^Georeferenced raster (1 km resolution) available for download and visualization at EarthEnv (https://www.earthenv.org/topography) under CC BY 3.0 license [45].

The influence of the aforementioned climatic and physiographical features was assessed by applying multivariate linear mixed models to the climatic data. A first set of linear mixed models was constructed to analyse the long-term variation of the response variable and the influence of a number of physiographical features (detailed in Table 2) and El Niño–Southern Oscillation (ENSO). The climatic forecast indices and climatic factors of interest were considered as the response variable, while the physiographic variables and the Multivariate ENSO Index (MEI) were included as explanatory variables. The variable “time” was included to account for the long-term variation in time-series data, whereas the moment of the year was defined using two sinusoidal components (sine and cosine) to consider the presence of a seasonal pattern [46]. The nested random factor “1 + time | Station ID” was included to account for the lack of independence of repeated measures, allowing both intercepts and “time” slopes to differ between meteorological stations.

In order to analyse whether the general pattern of long-term variation differs in consideration of each physiographical feature, we constructed a second set of models, which added the double interaction between “time” and each of the aforementioned explanatory variables.

In both set of models, explanatory variables were standardized to account for their different scales. Initial full models were constructed once with the variable “dist2contour” and then with the variable “dist2slope” (see Table 2 for a description), as these variables are highly correlated (>0.8) and could not be included concomitantly in the same model to avoid multicollinearity (highly correlated variables are probably contributing most of the same information to the response variable) [47]. The initial full models were simplified using likelihood ratio tests by removing non-significant terms in a stepwise backward elimination manner [48]. After model simplification, each pair of models including “dist2contour” or “dist2slope” was compared with the second-order Akaike Information Criterion (AICc) to select the most parsimonious and better fitting the data (the one with lesser AICc value if ΔAICc > 2; or the simpler model if ΔAICc < 2).

### 2.5. Spatial and statistical analyses

All the necessary calculations, spatial analyses and statistics have been carried out with R Statistical Software (‘R: A language and environment for statistical computing’, version 4.2.2 [2022-10-31 ucrt], http://www.r-project.org) and RStudio 2022.02.3.492 (‘RStudio: Integrated development environment for R’, http://www.rstudio.com/). Results were considered statistically significant when *p-value* <0.05.

## 3. Results

**Fig 3:**
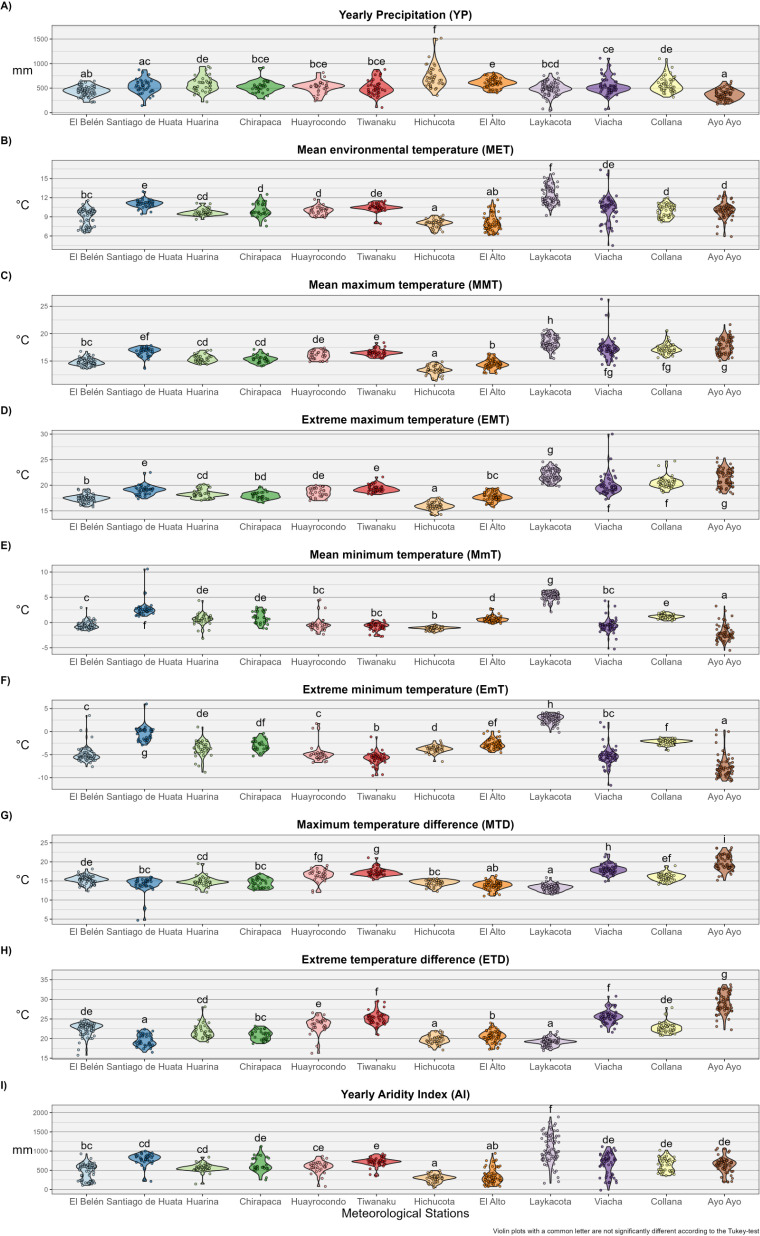
Violin plots summarizing yearly data for climatic factors recorded at a number of meteorological stations in the region of the Northern Bolivian Altiplano human fascioliasis hyperendemic area. Violin plots with a common letter are not significantly different according to the Tukey-test.

**Fig 4:**
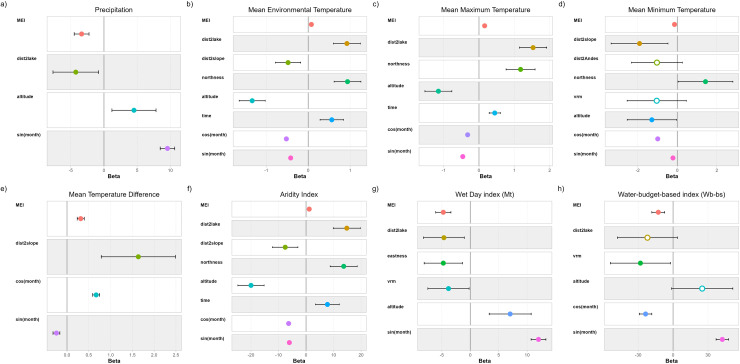
Model coefficients plots showing the influence of physiographical features and El Niño–Southern Oscillation (ENSO) on climatic factors and climatic forecast indices. Terms (y-axis) correspond to those in the best-approximating models. The x-axis displays model coefficients. Dots signify means and error bars 95% confidence intervals; filled dots depict significant coefficients (<0.05) and hollow dots depict non-significant coefficients. A coefficient overlapping with 0 signifies a neutral effect. Coefficients <0 and >0 signify negative and positive effects, respectively.

**Fig 5:**
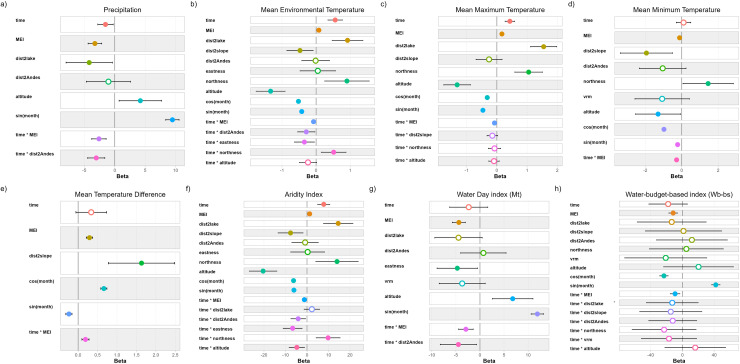
Model coefficients plots showing the long-term influence of physiographical features and El Niño–Southern Oscillation (ENSO) on climatic factors and climatic forecast indices. Terms (y-axis) correspond to those in the best-approximating models. The x-axis displays model coefficients. Dots signify means and error bars 95% confidence intervals; filled dots depict significant coefficients (<0.05) and hollow dots depict non-significant coefficients. A coefficient overlapping with 0 signifies a neutral effect. Coefficients <0 and >0 signify negative and positive effects, respectively.

Summarized climatic data from each of the meteorological stations studied are shown in Table 3, and violin plots summarizing yearly data are presented in Fig 3. Model selection and coefficients of selected models are presented in Tables 4 and 5 and Figs 4 and 5.

**Table 3:** Mean monthly values ± standard deviation, and (ranges) for climatic factors recorded at a number of meteorological stations in the Northern Bolivian Altiplano human fascioliasis hyperendemic area.

Variable	El Belén (1949–2017)	Santiago de Huata (1985–2020)	Huarina Cota Cota (1973–2011)	Chirapaca (1991–2020)	Huayrocondo (1991–2020)	Tiwanaku (1973–2016)
**MET (°C)**	8.9 ± 2.1 (2.1–15.6)	11 ± 1.4 (7–13.7)	9.7 ± 1.4 (5–13)	10.1 ± 1.7 (4.5–13.9)	9.9 ± 1.6 (4.3–14)	10.4 ± 1.9 (3.4–14.4)
**MMT (°C)**	14.8 ± 1.3 (11.1–25.9)	16.9 ± 1 (13–19.7)	15.4 ± 1.2 (11.3–18.7)	15.3 ± 1.2 (11.4–19.2)	16.1 ± 1.3 (11.8–20.2)	16.5 ± 1.4 (11.7–19.9)
**MmT (°C)**	−0.6 ± 3.7 (−9.3–8.5)	2.3 ± 1.9 (−4.2–6.4)	0.5 ± 3.6 (−10.8–6.1)	0.9 ± 3.4 (−7–7.3)	−0.5 ± 3.8 (−8.2–6.1)	−0.8 ± 4.2 (−13–5.6)
**EMT (°C)**	17.4 ± 1.6 (13–28.9)	19 ± 1.1 (16–22)	18.2 ± 1.6 (14.2–30)	18 ± 1.6 (14.2–26)	18.7 ± 1.6 (14.1–24)	19.3 ± 1.8 (15–25.1)
**EmT (°C)**	−5.2 ± 4.4 (−15.6–5)	−0.6 ± 2.7 (−9–4.3)	−3.7 ± 4.6 (−16.9–4.8)	−2.9 ± 3.9 (−12–4.7)	−5 ± 4.4 (−13.9–4)	−5.8 ± 5.3 (−19.6–3.1)
**MTD (°C)**	15.4 ± 3.6 (7.2–34.1)	14.5 ± 1.8 (10.4–20.8)	14.9 ± 3.4 (7.9–26.1)	14.4 ± 3.2 (7.6–22)	16.6 ± 3.7 (8.6–25.3)	17.2 ± 3.9 (9.3–30.8)
**ETD (°C)**	22.6 ± 4.2 (11.5–44.5)	19.6 ± 2.7 (15–28)	21.9 ± 4.3 (12.5–36.9)	20.9 ± 3.7 (12–31.5)	23.6 ± 4.1 (13–32.9)	25.1 ± 4.8 (14.2–39.5)
**Pt (mm)**	37.6 ± 37.2 (0–197.5)	46.4 ± 45.2 (0–214.8)	49.3 ± 51.1 (0–267)	44.1 ± 47.3 (0–300.2)	46 ± 48.3 (0–260.8)	43.6 ± 47.2 (0–246.3)
**YP (mm)**	444 ± 97.7 (211–647)	553.6 ± 121.6 (338–873)	592 ± 142.5 (359–942)	523 ± 118 (285–918)	518 ± 120.4 (240–816)	519 ± 158.7 (85–880)
**MP (mm)**	119 ± 32.4 (58–198)	142 ± 36.6 (74–215)	153 ± 45.3 (87–267)	144 ± 43.8 (81–300)	144 ± 44.7 (81–261)	136 ± 48.8 (31–246)
**DP (days)**	6 ± 6 (0–29)	6 ± 5.4 (0–26)	6 ± 6.2 (0–27)	6 ± 6.9 (0–27)	7 ± 7.4 (0–28)	6 ± 6.7 (0–28)
**DF (days)**	14 ± 11.6 (0–31)	3 ± 9.7 (0–31)	7 ± 11.8 (0–31)	8 ± 11.8 (0–31)	14 ± 12.1 (0–31)	14 ± 12.1 (0–31)
**AI (mm)**	39.6 ± 27.2 (−23.5–154.5)	68.6 ± 23.7 (11.8–121.5)	47.9 ± 21.4 (−6.1–107)	54.1 ± 25.9 (−9.9–124.5)	52.5 ± 24.4 (−11.3–114.6)	60.7 ± 29.1 (−17–136.6)
**Mt**	41.1 ± 46.9 (0–266.1)	33.2 ± 41 (0–246.8)	46.3 ± 56.5 (0–356.3)	43.2 ± 54.2 (0–415.4)	51.7 ± 62.8 (0–394.5)	41.1 ± 53.5 (0–338)
**Wb-bs**	35.4 ± 58.1 (0–483.9)	54.5 ± 102.9 (0–537.4)	61.2 ± 92 (0–617.2)	51.4 ± 85 (0–516.1)	66.4 ± 106.7 (0–770.7)	57.3 ± 108.1 (0–845.6)
**cumWb-bs**	92.9 ± 156.6 (0–812.3)	103.3 ± 218.6 (0–1178.5)	169 ± 288.3 (0–1465.1)	127.6 ± 224.2 (0–960.6)	148.2 ± 251 (0–1031.2)	124.3 ± 257 (0–1589.6)
**Variable**	**Hichucota (1979–2020)**	**El Alto (1962–2020)**	**Laykacota (1945–2020)**	**Viacha (1965–2015)**	**Collana (1973–2020)**	**Ayo Ayo (1958–2020)**
**MET (°C)**	7.9 ± 1.1 (3.5–10.4)	8.1 ± 2.1 (2–13.4)	12.5 ± 1.9 (7.2–18.1)	10.2 ± 2.5 (1.9–15.3)	10 ± 1.8 (4.2–14.7)	10 ± 2.5 (−3.2–15.2)
**MMT (°C)**	13.4 ± 1.1 (10.2–17.1)	14.5 ± 1.4 (7.2–19.4)	18.5 ± 1.7 (13.1–23.3)	17.1 ± 1.6 (11.8–22.7)	17.2 ± 1.6 (13.5–23.4)	17.7 ± 2.1 (8.5–24.7)
**MmT (°C)**	−1.1 ± 2.1 (−6.8–2.8)	0.7 ± 2.8 (−6.2–5.5)	5.3 ± 1.9 (−1.7–8.8)	−0.8 ± 4.3 (−11.6–6.1)	1.2 ± 2.8 (−5–6)	−2.2 ± 5.3 (−15.3–6.1)
**EMT (°C)**	15.9 ± 1.4 (11.5–20)	17.8 ± 1.7 (13.2–23)	22 ± 1.9 (16–27.2)	19.8 ± 1.9 (14–28)	20.5 ± 1.9 (15.5–27.5)	21.3 ± 2.6 (12–32)
**EmT (°C)**	−3.8 ± 2.8 (−12–1.5)	−2.8 ± 3.5 (−12.6–3.2)	2.8 ± 2.2 (−6–7.3)	−5.5 ± 5.4 (−17.7–3.8)	−2.2 ± 3.5 (−11.1–4)	−7.9 ± 6.4 (−23–3.2)
**MTD (°C)**	14.5 ± 2.7 (8.6–20.7)	13.8 ± 3 (6.1–22)	13.2 ± 1.7 (8.7–17.7)	17.8 ± 4.1 (10.3–27.3)	16.1 ± 2.8 (9.4–24)	19.9 ± 5.1 (9.3–32.6)
**ETD (°C)**	19.7 ± 3.2 (11.5–29)	20.5 ± 3.4 (12.2–31.6)	19.1 ± 2 (12.8–25.1)	25.4 ± 4.9 (14.3–36)	22.7 ± 3.3 (15–38)	29.3 ± 6 (14.4–44)
**Pt (mm)**	61.5 ± 72.1 (0–362.2)	51 ± 50.3 (0–244.5)	42.4 ± 42.3 (0–222.2)	46.1 ± 49.4 (0–319.1)	47.7 ± 49.9 (0–279.8)	31.8 ± 35.3 (0–207)
**YP (mm)**	736 ± 241.4 (353–1518)	611 ± 101.2 (404–811)	485 ± 135.7 (42–803)	546 ± 156.7 (315–1109)	571 ± 153.4 (316–1100)	371 ± 104.9 (174–639)
**MP (mm)**	205 ± 59.3 (107–362)	156 ± 35.4 (92–244)	125 ± 33 (20–222)	146 ± 53.3 (80–319)	153 ± 48.1 (77–280)	106 ± 34.4 (32–207)
**DP (days)**	6 ± 8.5 (0–31)	9 ± 7.4 (0–31)	8 ± 6.9 (0–31)	7 ± 6.6 (0–28)	7 ± 6.7 (0–29)	5 ± 5.7 (0–28)
**DF (days)**	24 ± 9.8 (0–31)	7 ± 12.1 (0–31)	0 ± 2.1 (0–26)	12 ± 12.3 (0–31)	6 ± 11.9 (0–31)	19 ± 11.9 (0–31)
**AI (mm)**	23.9 ± 13 (−16.2–60)	28.4 ± 25.7 (−24.1–114.4)	94 ± 35.9 (13.2–223.7)	58.5 ± 35.7 (−24.5–156.1)	53.7 ± 28.5 (−12–141.8)	55.9 ± 34.8 (−32–145.9)
**Mt**	52 ± 86.4 (0–499)	60.7 ± 69.3 (0–423.2)	35 ± 45.3 (0–327)	41.8 ± 55.2 (0–381.4)	48 ± 58.2 (0–402.5)	28.4 ± 38.8 (0–324.6)
**Wb-bs**	25.9 ± 49.4 (0–387.4)	45.8 ± 65.8 (0–442.8)	44.8 ± 105.9 (0–721)	66.5 ± 134.6 (0–1353.1)	75.4 ± 116.9 (0–818)	34 ± 72 (0–461.1)
**cumWb-bs**	76.3 ± 173.9 (0–1107.3)	142.5 ± 215.3 (0–1271.1)	74.4 ± 198.9 (0–1772.9)	157.2 ± 346.9 (0–3052.2)	185 ± 321 (0–1766.6)	55.6 ± 135.4 (0–890.3)

MET, mean environmental temperature; MMT, mean maximum temperature; MmT, mean minimum temperature; EMT, extreme maximum temperature, EmT, extreme minimum temperature; MTD, maximum temperature difference; ETD, extreme temperature difference; Pt, precipitation; YP, yearly precipitation; MP, maximum precipitation; DP, number of days with precipitation; DF, number of days with freeze; AI, aridity index; Mt, wet-day index; Wb-bs, water-based-budget index; cumWb-bs, cumulated water-based-budget index.

**Table 4:** Second-order Akaike Information Criterion (AICc) and weights for the selection of simplified models from the first set of multivariate linear mixed models for the analysis of the influence of physiographical features and El Niño–Southern Oscillation (ENSO).

Initial models		
Model A: *response variable ~ MEI* + *dist2lake* + *dist2contour* + *dist2Andes* + *eastness* + *northness* + *VRM* + *altitude* + *time* + *cos(month)* + *sin(month)* + *(1* + *time | stationID)*		
Model B: *response variable ~ MEI* + *dist2lake* + *dist2slope* + *dist2Andes* + *eastness* + *northness* + *VRM* + *altitude* + *time* + *cos(month)* + *sin(month)* + *(1* + *time | stationID)*		
**Simplified models**	**AICc**	**Weights**
Precipitation model*: Pt ~ MEI* + *dist2lake* + *altitude* + *sin(month)* + *(1* + *time | stationID)*		
MET models:		
model A: *MET ~ MEI* + *dist2lake* + *dist2contour* + *northness* + *altitude* + *time* + *cos(month)* + *sin(month)* + *(1* + *time | stationID)*	26658	0.027
model B:* MET ~ MEI *+* dist2lake *+* dist2slope *+* northness *+* altitude *+* time *+* cos(month) *+* sin(month) *+* (1 *+* time | stationID)*	26651	0.973
MMT models: *MMT ~ MEI* + *dist2lake* + *northness* + *altitude* + *time* + *cos(month)* + *sin(month)* + *(1* + *time | stationID)*		
MmT models:		
model A: *MmT ~ MEI* + *cos(month)* + *sin(month)* + *(1* + *time | stationID)*	35734	0.131
model B:* MmT ~ MEI *+* dist2slope *+* dist2Andes *+* northness *+* VRM *+* altitude *+* cos(month) *+* sin(month) *+* (1 *+* time | stationID)*	35730	0.869
MTD models:		
model A: *MTD ~ MEI* + *dist2lake* + *dist2contour* + *cos(month)* + *sin(month)* + *(1* + *time | stationID)*	34605	0.067
model B:* MTD ~ MEI *+* dist2slope *+* cos(month) *+* sin(month) *+* (1 *+* time | stationID)*	34599	0.933
AI models:		
model A: *AI ~ MEI* + *dist2lake* + *dist2contour* + *northness* + *altitude* + *time* + *cos(month)* + *sin(month)* + *(1* + *time | stationID)*	63337	0.026
model B:* AI ~ MEI *+* dist2lake *+* dist2slope *+* northness *+* altitude *+* time *+* cos(month) *+* sin(month) *+* (1 *+* time | stationID)*	63330	0.974
Mt model*: Mt ~ MEI* + *dist2lake* + *eastness* + *VRM* + *altitude* + *sin(month)* + *(1* + *time | stationID)*		
Wb-bs model*: Wb-bs ~ MEI* + *dist2lake* + *VRM* + *altitude* + *cos(month)* + *sin(month)* + *(1* + *time | stationID)*		

MET, mean environmental temperature; MMT, mean maximum temperature; MmT, mean minimum temperature; MTD, maximum temperature difference; Pt, precipitation; AI, aridity index; Mt, wet-day index; Wb-bs, cumulated water-based-budget index; dist2lake, distance to the Lake Titicaca; dist2slope, distance to slopes greater than 15 degrees representing inter-corridor hills; dist2Andes, distance to slopes greater than 25 degrees referring the steepest hillsides of the Oriental Andean Chain.

**Table 5:** Second-order Akaike Information Criterion (AICc) and weights for the selection of simplified models from the second set of multivariate linear mixed models for the analysis of the long/term variation of the influence of physiographical features and El Niño–Southern Oscillation (ENSO).

Initial models		
Model A: response variable ~ time*MEI + time*dist2lake + time*dist2contour + time*dist2Andes + time*eastness + time*northness + time*VRM + time*altitude + *cos(month)* + *sin(month)* + *(1* + *time | stationID)*		
Model B: response variable ~ time*MEI + time*dist2lake + time*dist2slope + time*dist2Andes + time*eastness + time*northness + time*VRM + time*altitude + *cos(month)* + *sin(month)* + *(1* + *time | stationID)*		
**Simplified models**	**AICc**	**Weights**
Precipitation model: Pt ~ time*MEI + *dist2lake* + time*dist2Andes + *altitude* + *sin(month)* + *(1* + *time | stationID)*		
MET models:		
model A: MET ~ time*MEI + *dist2lake* + *dist2contour* + time*dist2Andes + time*eastness + time*northness + time*altitude + *cos(month)* + *sin(month)* + *(1* + *time | stationID)*	26650	0.032
model B: MET ~ time*MEI +* dist2lake *+* dist2slope *+ time*dist2Andes + time*eastness + time*northness + time*altitude +* cos(month) *+* sin(month) *+* (1 *+* time | stationID)*	26643	0.968
MMT models:		
model A: MMT ~ time*MEI + *dist2lake* + time*dist2contour + time*eastness + *northness* + time*altitude + *cos(month)* + *sin(month)* + *(1* + *time | stationID)*	22726	0.039
model B: MMT ~ time*MEI +* dist2lake *+ time*dist2slope + time*northness + time*altitude +* cos(month) *+* sin(month) *+* (1 *+* time | stationID)*	22720	0.961
MmT models:		
model A: MmT ~ time*MEI + *cos(month)* + *sin(month)* + *(1* + *time | stationID)*	35698	0.120
model B: MmT ~ time*MEI +* dist2slope *+* dist2Andes *+* northness *+* VRM *+* altitude *+* cos(month) *+* sin(month) *+* (1 *+* time | stationID)*	35694	0.880
MTD models:		
model A: MTD ~ time*MEI + *dist2lake* + *dist2contour* + *cos(month)* + *sin(month)* + *(1* + *time | stationID)*	34589	0.070
model B: MTD ~ time*MEI +* dist2slope *+* cos(month) *+* sin(month) *+* (1 *+* time | stationID)*	34584	0.930
AI models:		
model A: AI ~ time*MEI + time*dist2lake + *dist2contour* + time*dist2Andes + time*eastness + time*northness + time*altitude + *cos(month)* + *sin(month)* + *(1* + *time | stationID)*	63321	0.022
model B: AI ~ time*MEI + time*dist2lake +* dist2slope *+ time*dist2Andes + time*eastness + time*northness + time*altitude +* cos(month) *+* sin(month) *+* (1 *+* time | stationID)*	63314	0.978
Mt model: Mt ~ time*MEI + *dist2lake* + time*dist2Andes + *eastness* + *VRM* + *altitude* + *sin(month)* + *(1* + *time | stationID)*		
Wb-bs models:		
model A: Wb-bs ~ time*MEI + time*dist2lake + time*dist2contour + time*dist2Andes + time*northness + time*VRM + time*altitude + *cos(month)* + *sin(month)* + *(1* + *time | stationID)*	91511	0.236
model B: Wb-bs ~ time*MEI + time*dist2lake + time*dist2slope + time*dist2Andes + time*northness + time*VRM + time*altitude +* cos(month) *+* sin(month) *+* (1 *+* time | stationID)*	91509	0.764

MET, mean environmental temperature; MMT, mean maximum temperature; MmT, mean minimum temperature; EMT, extreme maximum temperature, EmT, extreme minimum temperature; MTD, maximum temperature difference; ETD, extreme temperature difference; Pt, precipitation; PET, potential evapotranspiration; AI, aridity index; Mt, wet-day index; Wb-bs, cumulated water-based-budget index; dist2lake, distance to the Lake Titicaca; dist2slope, distance to slopes greater than 15 degrees representing inter-corridor hills; dist2Andes, distance to slopes greater than 25 degrees referring the steepest hillsides of the Oriental Andean Chain.

The violin plots and analysis of variance show certain heterogeneity among the meteorological stations (Fig 3), but limited to a narrow range of values, and thus a clear pattern is not completely discernible. However, the use of multivariate linear mixed models allowed to find a number of associations between climatic factors and geographical features.

The climatic factors assessed present a seasonal pattern, as evinced by the significant association with at least one of the sinusoidal components added to the linear mixed models to consider the presence of seasonality (Fig 4). An evident seasonality is observed in precipitation, majorly concentrated between November and April in most of the meteorological stations valued, and evinced by its high standard deviation (Table 3). Yearly precipitation averages 527 mm, with minimum values in Ayo Ayo (mean YP = 367 mm) and higher values in Hichucota (mean YP = 749 mm) (Table 3). Mean and maximum temperatures are somehow stable throughout the year, with mean temperatures barely reaching or surpassing the 10 °C transmission threshold (MET averages 9.9 ± 1.8 °C; MMT averages 16.1 ± 1.4 °C; Table 3). Minimum temperatures are more variable, with a minor decrease during April-May to August-September, signalled by the presence of freeze days (MmT averages 0.4 ± 3.3 °C; Table 3).

Since the calculation of the climatic forecast indices relies on precipitation, a seasonal pattern is found in the linear mixed models. The mean yearly value of Wb-bs index is higher than 1000 in almost every location (El Belén being an exception), while the maximum monthly value ever recorded surpasses the value of 800 in every location (El Belén presents the lower maximum monthly value, with 812) (Table 3).

Precipitation presents a negative and significant association with MEI and distance from Lake Titicaca (Fig 4a), decreasing with incrementing values of these fixed variables. Altitude presents a positive association with precipitation (Fig 4a). In the long-term, precipitation increased near the Oriental Andean Chain, but decreased farther from it (Fig 5a). Concerning the long-term influence of ENSO, precipitation increased when MEI presented negative values, and decreased with increasing values of MEI (Fig 5a).

In the long-term, mean and maximum temperatures increased significantly (Fig 4b and 4c), while minimum temperature and temperature amplitude were not affected (Fig 4d and 4e). MEI was positively associated to mean and maximum temperatures and temperature amplitude (Fig 4b, 4c and 4e), but presented a negative association with minimum temperatures (Fig 4d). Mean and maximum temperatures increased farther from Lake Titicaca (Fig 4b and 4c), while minimum temperatures and temperature amplitude seem not affected (Fig 4d and 4e). Mean and minimum temperatures were positively influenced by the proximity to minor hills nearby of the meteorological stations (Fig 4b and 4d), while temperature amplitude increased farther from the aforementioned elevations (Fig 4e). Temperature was positively associated with northness and negatively related with altitude (Fig 4b, 4c and 4d).

The long-term increment of mean and maximum temperatures was greater at declining values of MEI (Fig 5b and 5c), while minimum temperatures increased with decreasing values of MEI but decreased when MEI increased (Fig 5d). Concerning mean temperatures, its long-term variation was greater in the proximities of the Oriental Andean Chain and with northern and western exposition (Fig 5b). In the long-term, temperature amplitude was positively associated with MEI values (Fig 5e).

The aridity index increased significantly during the period assessed (Fig 4f). This index presents a positive association with MEI, with the distance from Lake Titicaca and with northness (Fig 4f). It decreases farther from the inner elevations and at increasing altitudes (Fig 4f). The long-term increment of the aridity index is more evident at lower values of MEI, nearer to the Oriental Andean Chain, with northern and western exposition, and at lesser altitudes (Fig 5f).

Concerning the climatic forecast indices, a long-term variation is not evident in the first set of models (Fig 4g and 4h). Both forecast indices present a significant and negative association with MEI and VRM (Fig 4g and 4h). Furthermore, the Mt index presents a negative association with the distance from the Lake Titicaca and eastness, and increases at higher altitudes (Fig 4g). Despite a long-term variation was not evident in the first set of models, both climatic forecast indices decreased in the long-term at increasing values of MEI. In the long-term, the Mt index increased in the proximities of Oriental Andean Chain, but decreased farther from it (Fig 5g).

## 4. Discussion

The complexity of fascioliasis transmission [22,27,49–54], the severe but usually neglected long-term consequences of its chronic infection [4–6], and the marked impoverishment of the populations affected [12], urged for the implementation of massive treatments within preventive chemotherapy strategies complemented with a One Health approach to tackle the critical situation of the human and animal fascioliasis hyperendemic area in the Northern Bolivian Altiplano [19]. In order to contribute to this multidisciplinary One Health action, the present study constitutes an unprecedented effort to analyse the influence of physiography on the long-term evolution of climatic factors and its impact on the transmission of fascioliasis, particularly focused on this high-altitude hyperendemic area.

The strong influence of the factors herein analysed on the transmission of fascioliasis are related to the life cycle characteristics of *F. hepatica*. Briefly, the life cycle of this parasite is strongly dependant of environmental features and mainly requires (i) temperatures above 10 °C enabling the development and maturation of its free-life and intra-molluscan stages [55,56] and the reproduction of lymnaeid snails [57,58], and (ii) the presence of suitable freshwater collections allowing the survival of lymnaeid snails and of the infective encysted metacercariae [17,54].

### 4.1. Climatic factor seasonality

A clear seasonal pattern is observed in precipitation, with a wet season centred on January and extending from November to April [59,60], and mostly related to changes in the zonal wind in the middle and upper troposphere over the central Andes [61]. On the other hand, seasonality is less evident in temperature. Maximum temperatures have only a weak annual cycle, while minimum temperature exhibits a more pronounced annual cycle [60]. This moderate seasonal variation in temperature seems to be related to the fact that solar radiation varies less than 30% from winter to summer [61]. Since the calculation of the fascioliasis climatic forecast indices relies on precipitation, the seasonality is evident. However, it must be considered that fascioliasis transmission in this hyperendemic high-altitude area does not solely rely on precipitation, but largely on the availability of permanent water sources [17,54]. Thus, temperature seems to be a more relevant factor than precipitation. Indeed, when analysing the fascioliasis forecast indices (i.e., maximum monthly value and mean yearly values accumulated during an entire year), the transmission threshold is surpassed, and nearly duplicated, in almost every location, indicating that transmission is feasible throughout the entire year.

### 4.2. Distance from the Lake Titicaca

As previously highlighted [17], the distance to Lake Titicaca exerts certain influence over the climatic factors under study. As expected due to the continentality effect of a proximal large body of water [62], the proximity to Lake Tititaca affects the records of temperature, buffering the mean and maximum temperatures in the localities under its influence. Thus, the localities farther from the lake present higher temperatures. The former has been already reported [59], and a similar effect was described in areas near to Lake Michigan, USA [63], reporting that closeness to the lake has a cooling effect in the summer and warming in the winter of the daily maximum temperature. Despite a warming effect on minimum temperature has been reported for areas closer to Lake Titicaca (reflected by the percentage of frost days) [60], we found that minimum temperatures are not influenced by the proximity to the Lake Titicaca (in coincidence with results concerning Lake Michigan by Im et al. [63]). However, it should be considered that higher maximum temperatures result in a greater evapotranspiration and, therefore, the aridity index increases with the distance from the Lake Titicaca, and there is a shorter permanency of temporary sources of water in localities distant from the Lake Titicaca. At any rate, the fascioliasis forecast indices do not appear to be influenced by the distance to the Lake Titicaca.

### 4.3. Closeness to nearest hills

Although no clear geographic patterns were found in the spatial distribution of minimum temperatures in the Altiplano of Bolivia and Peru [26], we found that minimum temperatures are related to the closeness to hills. Those localities distant from hills present lower mean and minimum temperatures and wider temperature differences. The influence of this kind of local factors and microclimate on the magnitude and temporal evolution of minimum temperatures in the Altiplano of Bolivia and Peru has already been suggested [26]. Although the closeness to hills has no influence on the fascioliasis climatic forecast indices, a positive influence on transmission is expected. Higher mean and minimum temperatures and narrower temperature amplitude in the proximities of hills may enhance the activity of lymnaeid populations, favouring their reproduction and survival. Further, these temperature increments may also enhance the maturation rate of the developmental stages of *F. hepatica*.

### 4.4. Distance from the Oriental Andean Chain

Our analyses suggest that the distance from the Oriental Andean Chain is not a determinant influence to the macro-climatic factors assessed, nor to the climatic forecast indices, in the human fascioliasis hyperendemic area. This lack of association with the closeness to the Oriental Andean Chain suggests that the strong local and regional differences in climate attributed to the presence of the Andes [26] might be largely attributed to altitude and hence to the increasing slopes of the respective mountainous foothills of this Chain (see below). However, the proximity to the Oriental Andean Chain is determinant when considering its influence on the availability of different sources of permanent water enabling fascioliasis transmission, as it has already been highlighted before [17,54].

### 4.5. Topographical features

In the fascioliasis hyperendemic area of the Northern Bolivian Altiplano, increasing altitude positively affects precipitation and logically leads to a negative influence on temperature. The former means that rain increases and temperature decreases at higher altitudes. This is in line with what is known for climate variability at high altitudes [64]: (i) high elevations sites are affected by mountain-induced orographic lift or convective instability that lead to regionally enhanced precipitation, and (ii) temperature decreases with elevation at a rate of about 6°C/km, although this is variable.

We found that temperature and the aridity index present a positive association with northness. Northness is derived from the topographical features aspect and slope [45]. In the northern hemisphere, a northness value close to 1 corresponds to a northern exposition on a vertical slope (i.e., a slope exposed to a very low amount of solar radiation), while a value close to −1 corresponds to a very steep southern slope, exposed to a high amount of solar radiation [45]. An opposite association with solar radiation is anticipated in the southern hemisphere: higher solar radiation with a northness value of 1 and a lower amount when northness reaches −1. Our results indicate that temperature and aridity increase with incrementing values of northness, meaning that northern exposition favours the aforementioned climatic factors. Given that Bolivia is located in the southern hemisphere, this is most probably due to the augmented solar radiation in slopes majorly faced to the north.

Our results indicate that the magnitude of the climatic forecast indices decreased with increasing values of the VRM. The VRM, a measure of the terrain profile and surface heterogeneity, quantifies local variation of slope [65], and ranges from 0 in flat regions to 1 in rugged ones [45]. This suggests that a flat and homogeneous terrain may enhance fascioliasis transmission, whether due to an increment on temperatures or by favouring the presence of freshwater collections.

### 4.6. Influence of El Niño—Southern Oscillation (ENSO)

The results obtained indicate that the Multivariate ENSO Index (MEI) presents a significant positive association with mean and maximum temperatures, temperature amplitude and aridity index, but a negative association with precipitation and minimum temperatures. This means that those periods characterized as El Niño (MEI values surpassing +0.5 °C) will result in dryer and hotter climatic conditions in the human fascioliasis hyperendemic area, with greater evapotranspiration. Conversely, the periods described as La Niña (MEI values below −0.5 °C) will result in rainier but colder climatic conditions in the area. These results agree with the traditional view of ENSO-precipitation relationships in a number of studies, which concluded that El Niño years (warm phase of ENSO) tend to be dry, while La Niña years (ENSO cold phase) are often associated with wet conditions on the Altiplano (e.g., [61,66,67]). However, opposite results have been described, stating that meteorological stations located near the Lake Titicaca and in the north-eastern Bolivian highlands showed more wet days, more very wet days, positive annual precipitation anomalies, and positive seasonal precipitation anomalies during El Niño than in La Niña years [68]. These results should be considered carefully, given the high complexity of the ENSO phenomenon evidenced by the use of different Southern Oscillation metrics (Niño 1+2, Niño 3, Niño 4, Niño 3+4, MEI) that show important differences, and contrasted effects in different South American regions [26,69,70].

Concerning fascioliasis transmission, the negative association of the MEI with the climatic forecast indices suggests that El Niño will decrease the risk of transmission, while La Niña might increase it. Yet, the opposite might occur. When exposed to dryer conditions, livestock will rely on the remnant sources of water available, which will be likely inhabited by thriving lymnaeid populations. Thus, transmission foci may become concentrated facilitating the disease transmission because of the need for both humans and livestock to draw on the same, less numerous freshwater sources. Such a situation has already been described for human fascioliasis in Argentina [71]. Furthermore, given the presence of lymnaeids in conditions to ensure the fascioliasis transmission, the developmental stages of the liver fluke depending on environmental features will be likely favoured by increasing temperatures.

### 4.7. Influence of physiographical features on the long-term variation of climatic factors and climatic forecast indices

Besides the already discussed effects, some of the physiographical features analysed in this study are influential due to the long-term evolution of the climatic change [25]. Although the precipitation evinced a general decline during the last decades in most of the fascioliasis hyperendemic area of the Northern Bolivian Altiplano [25], this pattern proved to be not homogeneous. Our results demonstrate a negative trend over time farther from the Oriental Andean Chain, but a positive one closer to it. Moreover, the magnitude of the warming process [25] is greater closer to the Oriental Andean Chain and in areas presenting northern and western exposition, which is accompanied by an increasing aridity.

In the long-term, the ENSO influence on precipitation and minimum temperatures is reinforced, strengthening its effect towards more extreme values. On the other hand, the long-term increment of mean and maximum temperatures was more evident with negative values of MEI, meaning that the warming process had been greater during La Niña than during El Niño events. These findings are consistent with evidence of stronger ENSO variability since the 1950s, which is expected to increase under greenhouse warning [72].

## 5. Concluding remarks

In general, the findings of this study revealed markedly heterogeneous climate characteristics throughout the endemic area, despite the apparent physiographic homogeneity of the endemic flatland corridors. This irregular pattern is influenced by physiographical features such as altitude, inner hills, closeness to Lake Titicaca, and El Niño–Southern Oscillation. These results highlight the importance of considering physiographical features outside but neighbouring the endemic area, an aspect usually not considered in studies dealing on the influences of climate and climate change on human and animal fascioliasis. It shows that an endemic area may climatically evolve differently in its various inner zones of the endemic area in question and emphasizes the need for continuous monitoring to assess whether control measures should be modified accordingly.
